# N-acetyl cysteine reverts the proinflammatory state induced by cigarette smoke extract in lung Calu-3 cells

**DOI:** 10.1016/j.redox.2018.03.006

**Published:** 2018-03-14

**Authors:** Ángel G. Valdivieso, Andrea V. Dugour, Verónica Sotomayor, Mariángeles Clauzure, Juan M. Figueroa, Tomás A. Santa-Coloma

**Affiliations:** a Institute for Biomedical Research (BIOMED, UCA-CONICET), Laboratory of Cellular and Molecular Biology, School of Medical Sciences, Pontifical Catholic University of Argentina (UCA) and The National Scientific and Technical Research Council of Argentina (CONICET), Alicia Moreau de Justo 1600, Buenos Aires C1107AFF, Argentina; b Fundación Pablo Cassará, Buenos Aires, Argentina

**Keywords:** ATP, adenosine triphosphate, cAMP, 3',5'-cyclic adenosine monophosphate, CF, cystic fibrosis, , cROS, cytoplasmic ROS, CFTR, cystic fibrosis transmembrane conductance regulator, mCx-I-III, mitochondrial NADH-cytochrome c oxidoreductase, COPD, Chronic obstructive pulmonary disease, CS, cigarette smoke, CSE, cigarette smoke extract, CTCF, corrected total cell fluorescence, DCFH-DA, 2´,7´-dichlorofluorescein diacetate, DMSO, dimethyl sulfoxide, EDTA, ethylenediaminetetraacetic acid, FBS, fetal bovine serum, HEPES, 4-(2-hydroxyethyl)− 1-piperazineethanesulfonic acid, IBMX, 3-isobutyl-1-methylxanthine, mCx-I, mitochondrial Complex I, MOPS, 3-(N-morpholino)propanesulfonic acid, mtROS, mitochondrial ROS, MTS, [3-(4,5-dimethylthiazol-2-yl)−5-(3-carboxymethoxyphenyl)−2-(4-sulfophenyl)−2H-tetrazolium, inner salt], NAC, N-acetyl cysteine, OXPHOS, oxidative phosphorylation system, PBS, phosphate buffered saline, PMSF, phenylmethylsulfonyl fluoride, ROS, reactive oxygen species, RQ, relative quantification, RT-qPCR, quantitative real-time RT-PCR, SPQ, 6-methoxy-N-[3-sulfopropyl]quinolinium, Cigarette smoke extract, Mitochondria, CFTR, ROS, COPD, Cystic fibrosis

## Abstract

Chronic obstructive pulmonary disease (COPD) and cystic fibrosis (CF) are lethal pulmonary diseases. Cigarette consumption is the main cause for development of COPD, while CF is produced by mutations in the *CFTR* gene. Although these diseases have a different etiology, both share a CFTR activity impairment and proinflammatory state even under sterile conditions. The aim of this work was to study the extent of the protective effect of the antioxidant N-acetylcysteine (NAC) over the proinflammatory state (IL-6 and IL-8), oxidative stress (reactive oxygen species, ROS), and CFTR levels, caused by Cigarette Smoke Extract **(**CSE) in Calu-3 airway epithelial cells. CSE treatment (100 µg/ml during 24 h) decreased *CFTR* mRNA expression and activity, and increased the release of IL-6 and IL-8. The effect on these cytokines was inhibited by N-acetyl cysteine (NAC, 5 mM) or the NF-kB inhibitor, IKK-2 (10 µM). CSE treatment also increased cellular and mitochondrial ROS levels. The cellular ROS levels were normalized to control values by NAC treatment, although significant effects on mitochondrial ROS levels were observed only at short times (5´) and effects on CFTR levels were not observed. In addition, CSE reduced the mitochondrial NADH-cytochrome c oxidoreductase (mCx I-III) activity, an effect that was not reverted by NAC. The reduced CFTR expression and the mitochondrial damage induced by CSE could not be normalized by NAC treatment, evidencing the need for a more specific reagent. In conclusion, CSE causes a sterile proinflammatory state and mitochondrial damage in Calu-3 cells that was partially recovered by NAC treatment.

## Highlights

• Cigarette smoke extract stimulates IL-6/IL-8 secretion in epithelial airway cells.
• The mechanism of IL-6/IL-8 induction involves NF-κB activation and ROS production.
• N-acetyl cysteine (NAC) treatment normalize cellular ROS and IL-6/IL-8 levels.
• Mitochondrial ROS levels and Complex I-III impairment were not normalized by NAC.
• Mitochondrial ROS scavengers might normalize the affected mitochondrial functions.

## Graphical abstract

**Figure:**
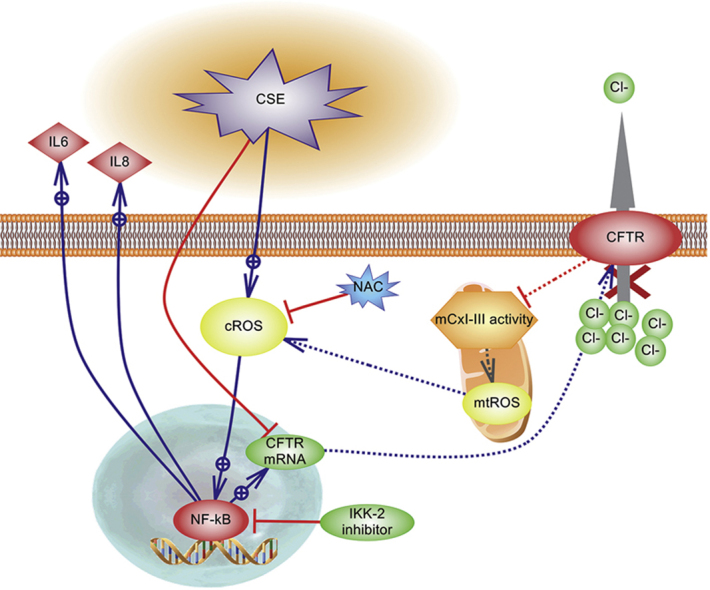

## 1 Introduction

Smoking, both active and passive, is the mayor non-infectious cause of respiratory disease and constitutes a risk factor for respiratory infections. Chronic obstructive pulmonary disease (COPD) is caused by environmental and genetic factors, and is characterized by chronic cough, respiratory secretions and progressive dyspnea and fibrosis, produced by chronic exposure of susceptible individuals to cigarette smoke (CS) [1]. The risk of disease increases proportionally to the amount of cigarettes smoked per day, although other factors, such as high exposure to dust, chemicals, and genetic factors (mutations in the α_1_-antitrypsin gene), could promote COPD [2].

Even though they are of different etiology, COPD shares similar respiratory symptoms with Cystic Fibrosis (CF) [1]. CF is an autosomal recessive disease [3] caused by mutations in the cystic fibrosis transmembrane conductance regulator (*CFTR*) gene, which encodes a chloride channel regulated by adenosine triphosphate (ATP) and 3´,5´-cyclic adenosine monophosphate (cAMP) [4], [5]. Both, COPD and CF, are characterized by airflow limitation, strong inflammatory response, recurrent infections, and progressive loss of lung function [1]. In the last years, several reports show that COPD is also associated with alterations in the CFTR channel function [1], [6], [7], [8], [9], [10], [11], [12]. Studies in CF suggest that the impairment of the CFTR is associated with an alteration of the mitochondrial function, in particular the mitochondrial complex I (mCx-I) activity and reactive oxygen species (ROS) production [13], [14], [15], [16], [17], [18]. The extent to which CF and COPD share similar pathological mechanisms could be of interest to identify new therapeutic targets for both diseases.

A common adverse characteristic of COPD and CF is a strong inflammatory response, initiated with the increased secretion of proinflammatory cytokines and ROS levels [1]. In the treatment of respiratory diseases, the antioxidant N-acetylcysteine (NAC) has emerged as a mucolytic, antioxidant and anti-inflammatory drug [19], [20]. NAC antioxidant effects occurs directly, through its free sulfhydryl group that serves as a source of reducing equivalents, and indirectly, through the replenishment of intracellular GSH levels [21]. In fact, NAC acts as a cysteine precursor for the synthesis of glutathione (GSH), increasing the antioxidant protection of the cells [22].

The aim of this work was to study the extent of the protective effect of the antioxidant N-acetylcysteine (NAC) over the proinflammatory state and oxidative stress caused by CSE [7], [19], [23]. For this purpose we used cultured Calu-3 airway cells as a model system, measuring the secretion of the cytokines IL-6 and IL-8 as proinflammatory markers [23]. Calu-3 cells express high levels of CFTR [24] and constitute a good *in vitro* model to study human respiratory function, inflammatory responses and diseases [25]. In cells exposed to CSE, we observed an increased IL-6 and IL-8 secretion induced through NF-κB activation, together with a reduced CFTR expression and activity. The reduction in the CFTR expression could not be reverted by NAC. However, the increased secretion of these cytokines was blocked by NAC, suggesting that ROS contributed to the NF-κB activation. We also demonstrated a fast induction of the mitochondrial ROS levels (mtROS) and a later mitochondrial NADH cytochrome c oxidoreductase (Complex I-III) activity impairment that could not be improved with NAC treatment. The NAC effects over ROS and cytokine levels suggest that an antioxidant treatment may help to reduce inflammation in COPD; it also evidences the need for an antioxidant therapy directed to specifically reduce the mitochondrial oxidative stress and damage to the oxidative phosphorylation system (OXPHOS) induced by CSE, which was not reverted with NAC treatment. On the other hand, the possible CFTR role in the proinflammatory response is not clear yet.

## 2 Materials and methods

### 2.1 Chemicals

Dimethyl sulfoxide (DMSO, culture grade), valinomycin, dibutyryl-cAMP, IBMX (3-isobutyl-1-methylxanthine), cytochrome c and (-)-isoproterenol hydrochloride were purchased from Sigma-Aldrich (St. Louis, MO). Trypsin was purchased from Life Technologies (GIBCO BRL, Rockville, MD) and SPQ (6-methoxy-N-[3-sulfopropyl]quinolinium) from Invitrogen (Carlsbad, CA). MitoSOX and 2´,7´-dichlorofluorescein diacetate (DCFH-DA) was from Molecular Probes (Eugene, OR). The IKK-2 inhibitor SC-514 (CAS 354812–17-2) was from Calbiochem (San Diego, CA). Cigarette smoke extract (CSE) (stock solution 40 mg/ml in DMSO) was from Murty Pharmaceuticals (Lexington, KY). N-acetylcysteine (NAC) (0.5 M stock solution in water (pH = 7.4)) was purchased from PharmaZell (PharmaZell, Chennai und Vizag, India). All other reagents were analytical grade. The stock solutions of valinomycin, IBMX, and dibutyryl-cAMP were prepared at 1000× in culture-grade DMSO. Isoproterenol was dissolved in water at 1000× concentration.

### 2.2 Cell culture

Calu-3 cells (ATCC Cat# HTB55), epithelial airway cells known to express wt-CFTR [24], were used in the experiments. Cells were cultured in DMEM (Life Technologies, GIBCO BRL, Rockville, MD) supplemented with 10% FBS (Life Technologies, GIBCO BRL, Rockville, MD), 100 U/ml penicillin, 100 µg/ml streptomycin, and 0.25 µg/ml amphotericin B (GIBCO BRL).

### 2.3 CSE exposure

Cells were incubated with CSE at the concentrations and times indicated for each assay. For viability assays, 10–200 µg/ml CSE were used for various times (5–72 h). For the rest of the experiments, cells were exposed to 100 µg/ml CSE during 24 h since this concentration and time did not negatively affect viability. Control cells contained the same final amount of DMSO (0.25%) [26] as in CSE exposed cells.

### 2.4 Cell viability and proliferation assays

Calu-3 cells were grown in 96-well plates (10,000 cells/cm^2^ in 100 µl of DMEM-10% FBS medium). The cells were incubated with different concentrations of CSE (10, 50, 100 and 200 μg/ml), or the vehicle (DMSO) at different times (0, 5, 24, 48 and 72 h). We used a commercial cigarette smoke extract (CSE) to assure the reproducibility of the assays. Cell viability was evaluated by using the CellTiter 96® AQueous One Solution Cell Proliferation Assay (Promega, Madison, WI), according to manufacturer´s instructions. Briefly, after washing with PBS, pH 7.4, cells were treated with staining solution containing the tetrazolium compound MTS [3-(4,5-dimethylthiazol-2-yl)−5-(3-carboxymethoxyphenyl)−2-(4-sulfophenyl)−2H-tetrazolium, inner salt] and an electron coupling reagent (phenazine ethosulfate; PES). Absorbance was recorded at 490 nm using a microplate reader (model Benchmark, Bio-Rad, Hercules, CA).

### 2.5 Quantitative real-time RT-PCR (RT-qPCR)

To determine *CFTR* mRNA expression levels, RT-qPCRs were performed and the ΔΔCt method was used for comparative quantification, as previously described [17], [27]. Briefly, total RNA (4 μg) from Calu-3 cells, treated with 100 µg/ml CSE or vehicle for 24 h, was used for reverse transcription by using M-MLV Reverse Transcriptase (Promega) and Oligo-dT, according to the manufacturer's instructions (100 U of RT/μg of RNA). PCR conditions were performed as previously described [17], [27]. qRT-PCR reactions were carried out in an Applied Biosystems 7500 Real-Time PCR equipment, and thermocycler conditions were: denaturation at 94 °C (5 min), and 40 cycles of 94 °C (30 s), 60 °C (30 s), and 72 °C (30 s).

### 2.6 CFTR transport activity in Calu-3 cells

The fluorescent probe SPQ was used to measure the CFTR chloride transport activity, as we previously described [17], [27], [28], with some modifications. Calu-3 cells were seeded at a density of 40,000 cells/cm^2^ in 2 ml of medium and grown at confluence in p6 wells plates containing one rectangular coverslips (22 × 8 mm, from Hitachi) pre-treated with a coating solution (10 µg/ml fibronectin, 4.4 µg/ml collagen, 1.5 µg/ml BSA in DMEM/F12). The cells were incubated with 100 μg/ml CSE or the vehicle for 24 h. Cells were loaded with SPQ by hypotonic shock by using 5 mM SPQ (dissolved 1:1 in serum-free DMEM/F12:H_2_O distilled and sterile, washed three times with NaI buffer (135 mM NaI, 10 mM Glucose, 1 mM CaSO_4_, 1 mM MgSO_4_, 10 mM HEPES, 2.4 mM K_2_HPO_4_, and 0.6 mM KH_2_PO_4_, pH 7.4)) and maintained in NaI at 37 °C for 30 min. The measurement of the CFTR activity was performed as previously described [28]. Data was plotted as F/Fi −1 vs time (F: fluorescence; Fi: initial fluorescence measured after the NaI buffer perfusion). The halide efflux slopes were estimated by using the initial 6 points of time after the CFTR response to stimulation started.

### 2.7 Secretion of IL-8 and IL-6

Calu-3 cells were seeded (27,000 cells/cm^2^ in 100 µl of DMEM-10% FBS medium) in 96 well-plates and grown to confluence. The cells were then incubated with 100 μg/ml CSE, 100 μg/ml CSE plus 10 μM IKK-2 inhibitor, 100 μg/ml CSE plus 5 mM NAC or the vehicle for 24 h. IL-6 and IL-8 were measured from supernatants using the human IL-6 and IL-8 ELISA set (BD OptEIA™ - Human IL-8 ELISA Set, and BD OptEIA™-Human IL-6 ELISA Set, BD Biosciences, San Diego, CA). Measurements were performed using a microplate reader according to manufacturer´s instructions (model Benchmark, Bio-Rad).

### 2.8 Cellular reactive oxygen species (ROS)

Cellular ROS levels were measured using the fluorescent probe DCFH-DA in cells cultured in 96-well plates (Greiner Bio-One, Germany; 655,090) as previously reported [13]. Briefly, 27,000 cells/cm^2^ were seeded and grown to confluence in DMEM − 10% FBS medium. Then, cells were incubated in Hank´s buffer containing 10 µM of DCFH-DA and incubated at 37 °C in a 5% CO_2_/air incubator for 40 min. Then, cells were washed five times with Hank´s buffer and treated for 1 h with DMSO as vehicle, 100 μg/ml CSE or 100 μg/ml CSE plus 5 mM NAC. The fluorescence was measured at different times to determine the maximal signal in a fluorescence plate reader (NOVOstar BMG LABTECH GmbH, Ortenberg, Germany) at 37 °C. Filters were Ex = 510 ± 10 nm, Em = 540 ± 10 nm and readings were performed by using 10 cycles (3 flashes per well and cycle; excitation and measurements were done from the bottom of the plate).

### 2.9 Confocal microscopy for cytoplasmic ROS measurement

To test the intracellular fluorescence signal and discard background, the DCF fluorescence was observed in a Zeiss LSM 510 confocal microscope (Plan-Neofluar 100×/1.3 Oil objective) (Carl Zeiss, Jena, Germany). Briefly, Calu-3 cells were seeded (27,000 cells/cm^2^) on chambered coverglasses (4-chamber, Nunc, Cat. No. 155383, Lab-Tek, Thermo Fisher Scientific, Rochester, NY) and cultured in DMEM-10% FBS medium. Cells were incubated in Hank´s buffer containing 10 µM of DCFH-DA and incubated at 37 °C in a 5% CO_2_/air incubator for 40 min. Then, cells were washed with 0.4 ml of Hank´s buffer five times and incubated with vehicle, 100 μg/ml CSE or 100 μg/ml CSE plus 5 mM NAC for 1 h. Cell images were acquired by using a LSM 510 Zeiss confocal microscope with a laser line of 488 nm and a long-pass filter LP505. The detector gain, offset, laser potency and pinhole were maintained for all conditions to compare the florescence intensity.

### 2.10 Confocal microscopy for mitochondrial ROS measurement

To measure mtROS, cells were cultured as above indicated for DCFH-DA, changing the probe to MitoSOX (5 µM), and incubating at 37 °C in a 5% CO_2_/air incubator for 10 min. Then, cells were washed with Hank´s buffer four times (one minute each) and incubated in 100 µl of Hank's buffer. *In vivo* time-lapse images were acquired before the treatments with a final concentration of 100 μg/ml CSE or 100 μg/ml CSE plus 5 mM NAC. Confocal images were acquired in the LSM 510 confocal microscope by using a Plan-Neofluar 100×/1.3 Oil objective, a 488 nm laser line, and a long-pass (LP) filter of 560 nm (filter LP560). The detector gain, offset, laser potency and pinhole size were maintained for all conditions to compare the fluorescence intensity. ImageJ (http://imagej.nih.gov/) was used to quantify total fluorescence intensity by using corrected total cell fluorescence (CTCF) [26], [29], [30].

### 2.11 Mitochondria isolation

Mitochondria were isolated by using differential centrifugations as was previously described [13], [17], [31]. Briefly, cells were seeded at a density of 25,000 cells/cm^2^ (p100 dishes have 60 cm^2^) and cultured for 24 h in 5 ml DMEM- 10% FBS, at 37 °C in a humidified air atmosphere containing 5% CO_2_. Then, cells were washed with PBS, scrapped and centrifuged at 600×*g* for 5 min at 4 °C. The pellet was resuspended in isolation buffer (0.25 M sucrose, 25 mM MOPS, pH 7.4) and the cells were permeabilized by adding 0.12% w/v digitonin for 30 s on ice. The samples were diluted in three volumes of isolation buffer and centrifuged at 10,000×*g* for 20 min at 4 °C. The resultant pellet was resuspended in 500 µl of isolation buffer and centrifuged at 800×*g* for 10 min at 4 °C. The supernatant was centrifuged at 10,000×*g* for 20 min at 4 °C, and the mitochondrial pellet was resuspended in 10–20 µl of BN-sample buffer A (1 M aminocaproic acid, 50 mM bis-Tris-HCl, 10 μM pepstatin, 10 μM leupeptin, 100 μM PMSF, 1 mM EDTA, pH 7.0) [13], [17]. Mitochondrial protein concentration was measured by Lowry [32] by using aliquots of mitochondrial extract incubated with 0.1 N NaOH for 30 min at 37 °C, to dissolve mitochondrial membranes.

### 2.12 Spectrophotometric determination of mitochondrial NADH-cytochrome c reductase (mCx-I-III) activity

The NADH-cytochrome c reductase activity (mCx-I plus mCx-III) was spectrophotometrically measured in mitochondria from Calu-3 cells treated with 100 μg/ml CSE, 100 μg/ml CSE plus 5 mM NAC or vehicle for 24 h, in the presence/absence of rotenone (10 µM), as it was previously described [13], [33], [34]. Briefly, mitochondrial preparations were subjected to three freeze-thaw cycles to make them permeable to substrates. To measure the mCx-I-III activity, the mitochondria (equivalent to 100 µg of proteins) were resuspended in buffer solution (100 mM H_2_KPO_4_/HK_2_PO_4_, 0.5 mM KCN, 200 μM NADH, 25 μM oxidized cytochrome c, at pH 7.4). The reduction of cytochrome c was recorded by monitoring the increase in absorbance per minute for 2 min at 550 nm and 30 °C, and expressed as percentage, considering the activity in control cells as 100%. To determine the mCx-I activity, inhibition of NADH cytochrome c reductase activity by rotenone (10 µM) was measured in each sample after 5 min of preincubation with the inhibitor and the remaining values (insensitive to rotenone) were subtracted from the total activity.

### 2.13 Statistics

Unless otherwise indicated, all the assays were performed in duplicates and the experiments were repeated at least three times (n = 3). RT-qPCR reactions were carried out by using intra-assay triplicates. The final RT-qPCR quantification values were obtained as the means of the relative quantification (RQ) values for each independent experiment (n = 3). One-way ANOVA and Tukey's *post-hoc* test was applied to determine significant differences among samples (p < 0.05). All values are shown as mean ± SEM; the n value (number of replicates) is indicated in each case.

## 3 Results

### 3.1 Effects of CSE on Calu-3 cells viability

**Fig. 1:**
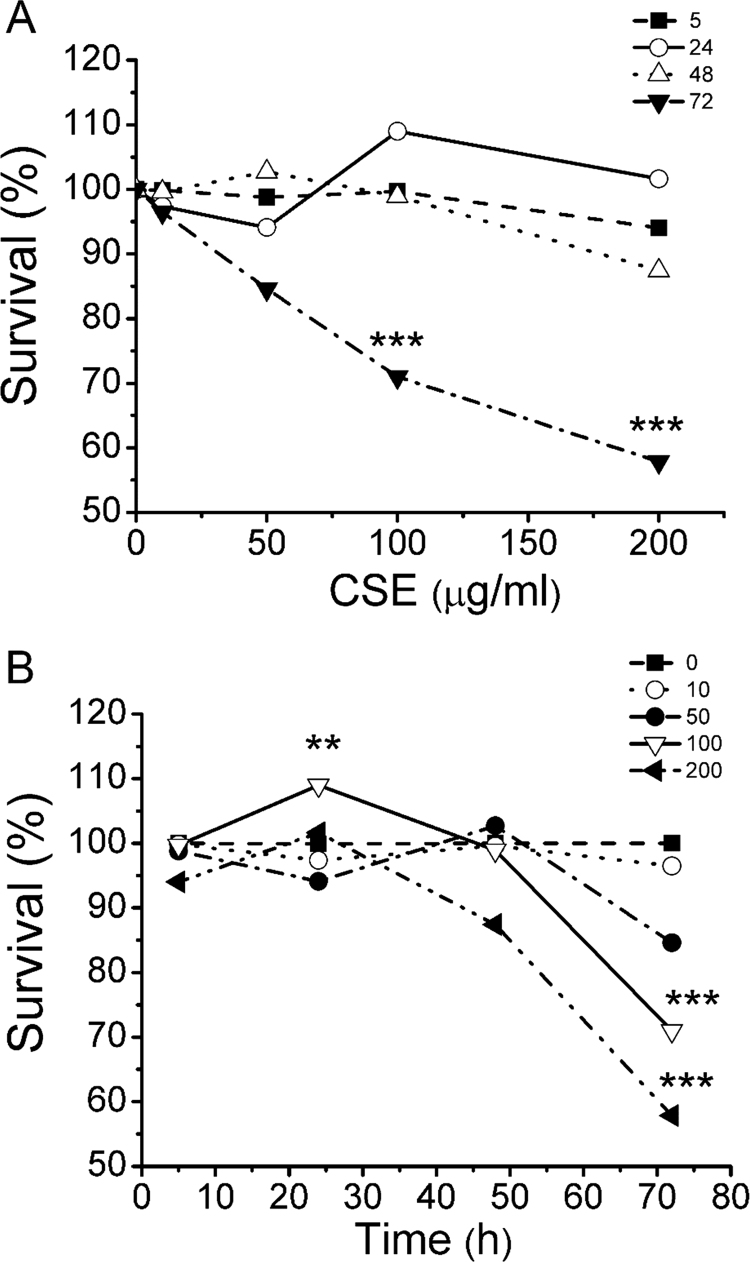
**Cell viability in Calu-3 cells exposed to CSE.** A: Dose-response cell viability for CSE exposure (0, 10, 50, 100, and 200 µg/ml) at different times (5 dash line, 24 solid line, 48 dot line and 72 dash dot line h). B: Survival vs time plot for CSE exposure at different CSE concentrations (0 dash line, 10 dot line, 50 dash dot line, 100 solid line, and 200 dash dot dot line µg/ml). Viability was expressed as survival percentage (%) referred to control cells without CSE treatment (vehicle) as 100%. ** indicates p < 0.01 and *** p < 0.001 compared to control (untreated) cells (n = 4). Statistical analyses were performed by ANOVA and Tukey's test.

First, we measured the effects of continuous exposure to several concentrations of CSE on Calu-3 cells viability. As shown in Fig. 1A, plotting survival (%) vs CSE concentration (µg/ml), cell viability was significantly diminished only after 72 h of exposure to 100 or 200 µg/ml (p < 0.001). Similarly, plotting survival (%) vs time (Fig. 1B), a significant inhibition was observed with 100 and 200 µg/ml CSE (~27% and ~35% respectively) after 72 h of exposure (p < 0.001). In view of these results, we decided to run further experiments with the sublethal concentration of 100 µg/ml CSE during 24 h. Treatments for 24 h with NAC (5 mM), or CSE (100 µg/ml) + NAC (5 mM), did not have effects on cell viability (Supplementary Fig. 1).

### 3.2 CSE affects CFTR expression and activity

**Fig. 2:**
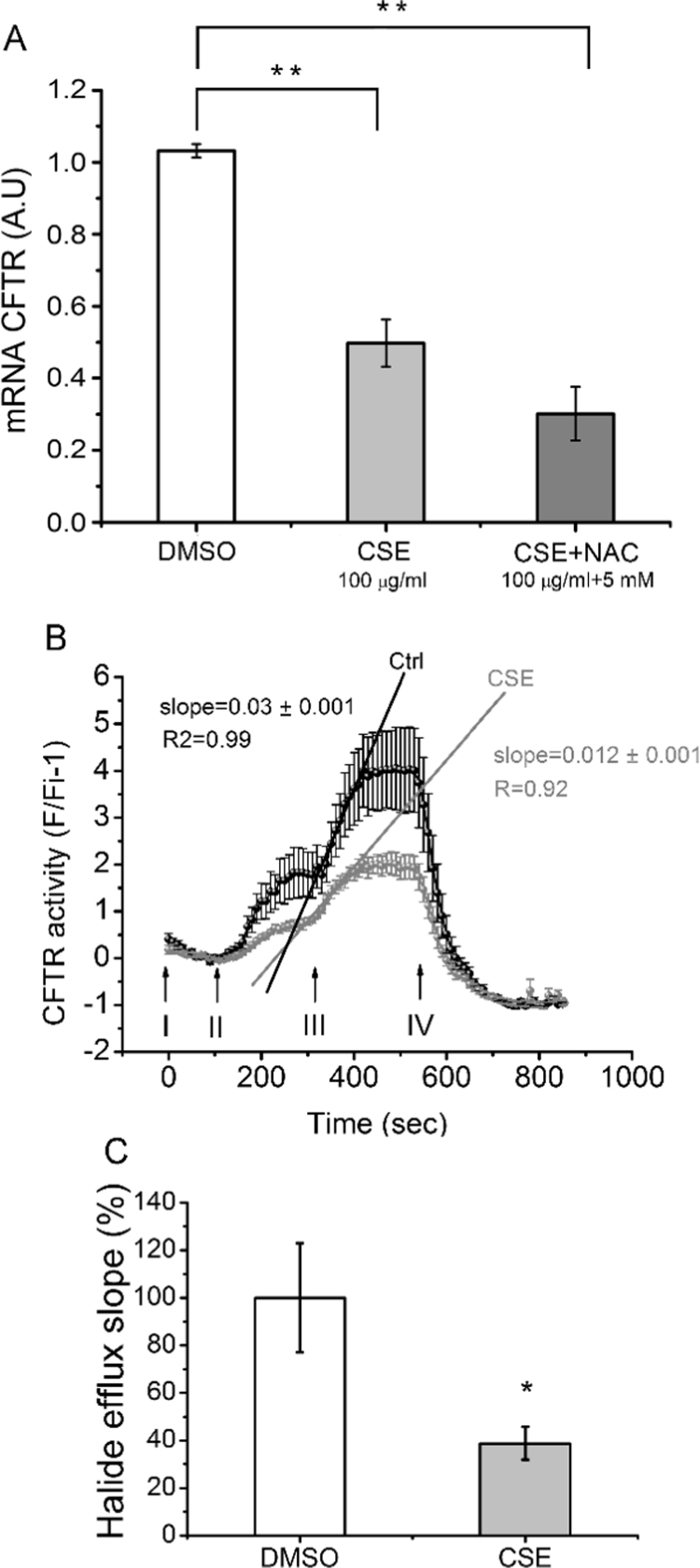
**CSE inhibit CFTR mRNA expression and activity.** A: *CFTR* mRNA levels in Calu-3 cells treated with 100 μg/ml CSE (Cigarette Smoke Extract) or DMSO (vehicle) for 24 h, measured by quantitative real-time RT-PCR (qRT-PCR). The results were expressed as arbitrary units (A.U.). Measurements correspond to four independent experiments (n = 4), each done in duplicate. B: CFTR channel halide transport activity was measured in Calu-3 cells treated with 100 μg/ml CSE or DMSO by using the SPQ Cl^-^ sensitive probe. Arrows indicate the times of buffers addition; I: NaI buffer, II: NaNO_3_ buffer, III: NaNO_3_ buffer + cAMP cocktail, IV: KSCN 150 mM + 5 μM valinomycin. Fluorescence values were calculated as F= (F-Fq)/(Fi-Fq) −1; Fi, are the initial fluorescence values in NaI buffer. Fq corresponds to background values (fluorescence quenching in the presence of KSCN+valinomycin). The graph is the mean of four independent experiments (n = 4). C: Halide efflux slopes, corresponding to SPQ fluorescence changes of Calu-3 cells incubated with CSE or DMSO. The slopes were calculated from the linear regressions of the first 6 points after CFTR stimulation and were plotted as percentage (%) relative to controls. All data were expressed as mean ± SEM. * * indicates p < 0.01 and * p < 0.05, as compared to control cells. Statistical analyses were performed by ANOVA and Tukey's test.

Low CFTR levels have been recently associated with COPD [1], [10]; therefore, we wanted to validate the use of Calu-3 cells as a model for COPD measuring *CFTR* mRNA expression and activity after CSE exposure. As shown in Fig. 2A, the *CFTR* mRNA in cells exposed to 100 μg/ml of CSE for 24 h was reduced (p < 0.001, n = 4) compared to control cells (vehicle). On the other hand, NAC had not effects on the CFTR mRNA levels decreased by CSE treatment (Fig. 2A).

Then, to test if the decreased *CFTR* mRNA expression caused by CSE was reflected in an impairment of the CFTR function, the activity of the channel was measured by using the chloride sensitive fluorescent probe SPQ. As shown in Fig. 2B, the CSE treatment decreased both the basal and the cAMP-stimulated chloride efflux. The halide efflux slopes, that reflects the activation state of the channel, also showed a lower CFTR activation in Calu-3 cells treated with CSE (slope = 0.012 ± 0.001; n = 3) compared to control cells (DMSO treated cells) (slope = 0.030 ± 0.001; n = 3). In Fig. 2C, the halide efflux slopes were expressed as percentage referred to control cells as 100%. Thus, cells treated with CSE showed a significant (p < 0.05) decrease, near to 60%, in the activity of the CFTR compared to control cells.

### 3.3 Effect of CSE on proinflammatory cytokines IL-6 and IL-8

To test if the cigarette smoke extract (CSE) induces a proinflammatory response in Calu-3 cells, the IL-6 and IL-8 secretion were measured on the cultured media after 24 h incubation. We also tested if the treatment with the commonly used antioxidant NAC could protect Calu-3 cells against the CSE oxidative response as occurs in other model systems [19], [23].

**Fig. 3:**
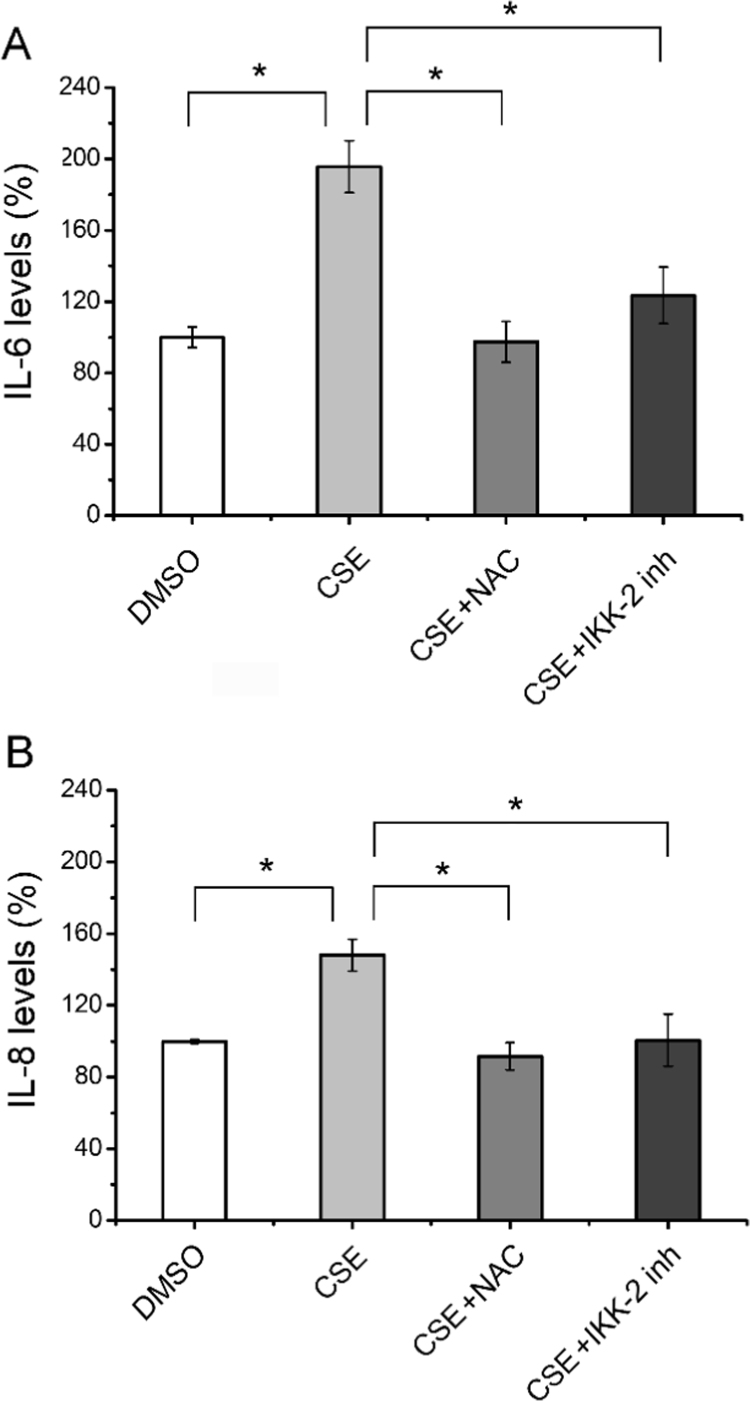
**CSE induces IL-8 and IL-6 secretion through NF-κB activation.** The effect of CSE on the proinflammatory cytokines IL-8 and IL-6 secretion was evaluated by ELISA in Calu-3 cells incubated with 100 μg/ml CSE (CSE) for 24 h. The role of the increased ROS production in the IL-6/IL-8 secretion was tested by incubating with 100 μg/ml CSE + 5 mM NAC (CSE+NAC). Calu-3 cells were incubated in presence of 100 μg/ml CSE and 10 µM IKK-2 inhibitor (CSE+IKK-2 inh) to block the NF-κB signal. A) IL-8 and IL-6 (B) secretion were plotted as percentage compared to control cells as 100% and expressed as mean ± SEM. * p < 0.05. Statistical analyses were performed by ANOVA and Tukey's test.

As shown in Figs. 3A and 3B, a significant (p < 0.01) increase in the IL-6 (3 A) and IL-8 (3B) secretion was observed in cells treated with CSE (100 µg/ml, 24 h) compared to control cells (vehicle treated cells). Noteworthy, the NAC treatment completely reverted the CSE effects, showing the treated cells similar levels of IL-6 and IL-8 to those observed in control cells. These data suggest that cytokine induction by CSE was caused by increased cellular ROS levels.

It is known that the expression of proinflammatory cytokines is regulated by the transcription factor NF-κB [35], [36], [37], and that its activation can be induced by increased ROS levels [38], [39], [40], [41], [42]. To test if the increased IL-6 and IL-8 secretion caused by CSE through ROS was mediated by the NF-κB, Calu-3 cells were incubated with CSE in presence of 10 µM of the NF-κB pathway inhibitor IKK-2. Co-treatment of CSE and IKK2 decreased IL-6 and IL-8 secretion compared to Calu-3 cells treated with CSE alone (Figs. 3A and 3B), suggesting that NF-κB is also involved in the activation by CSE of both cytokines, IL-6 and IL-8.

### 3.4 CSE increased cellular ROS

**Fig. 4:**
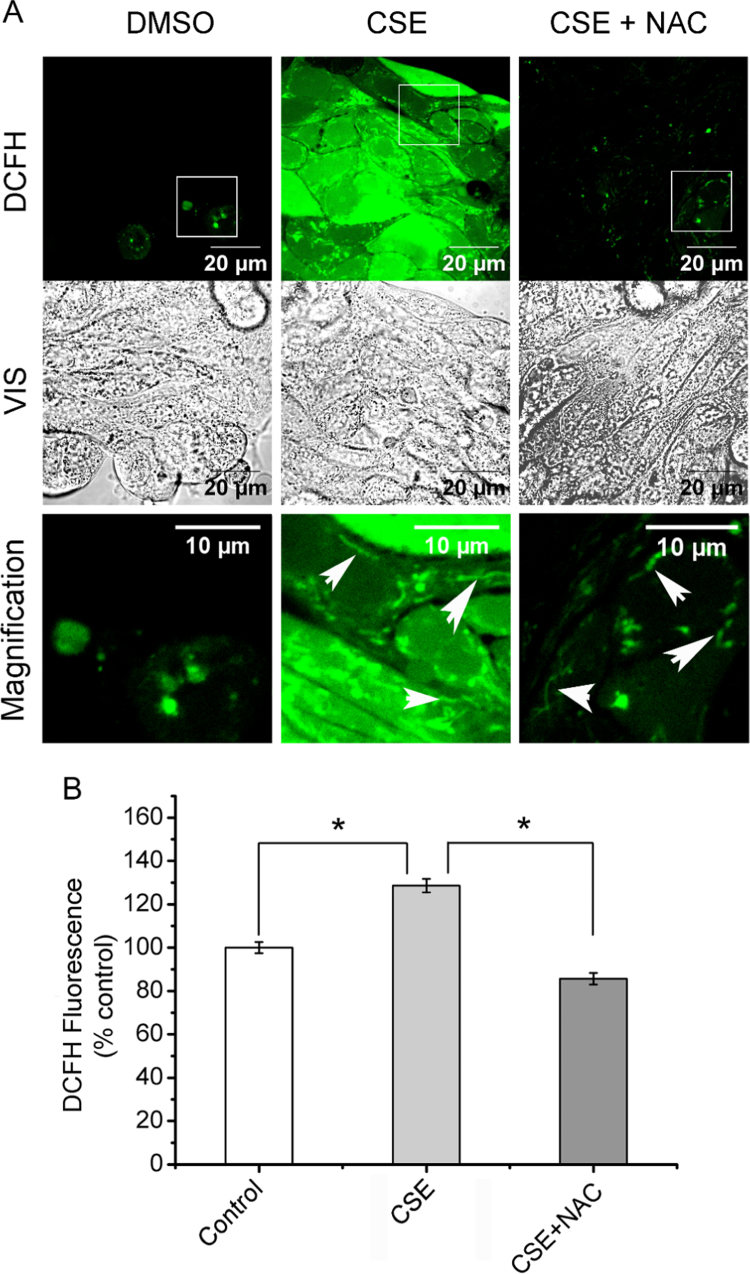
**Cellular ROS increase by CSE exposure and NAC protection.** A) Confocal image of total cellular ROS levels measured by using DCFH-DA, in cells treated with CSE or CSE+NAC for 1 h. Images were acquired by using a Plan-Neofluar 100 × /1.3 Oil objective, a laser line of 488 nm, and a LP filter of 505 nm. VIS indicates the visible (transmitted light) image of cells. Magnifications corresponded to the white squares indicated in the figures. Arrows indicates regions of intracellular accumulation of ROS. B: Spectrofluorometry of cellular ROS levels measured by using DCFH-DA in cells incubated with 100 μg/ml CSE (CSE), DMSO or 100 μg/ml CSE + 5 mM NAC (CSE+NAC) for 1 h.

Cellular ROS (cROS) levels were measured in cells exposed to CSE by using the fluorescent probe DCFH-DA. As shown in Fig. 4A, the fluorescence observed by confocal microscopy in cells exposed to CSE increased and was highly diffused throughout the cells. On the other hand, NAC (5 mM) completely blocked the CSE-induced cROS. Some remaining fluorescence was observed in NAC treated cells, indicated by arrows in Fig. 4A. To quantify these responses, the DCF fluorescence was measured by using a microplate reader. In agreement with the confocal images, the ROS levels were significantly increased in cells treated with CSE for 1 h compared to control cells (p < 0.05) (Fig. 4B). In addition, the presence of NAC 5 mM reduces the CSE-induced cROS (Fig. 4B). These results are in agreement with the response of IL-6 and IL-8 to CSE and NAC (Fig. 3).

### 3.5 CSE increased mitochondrial ROS

**Fig. 5:**
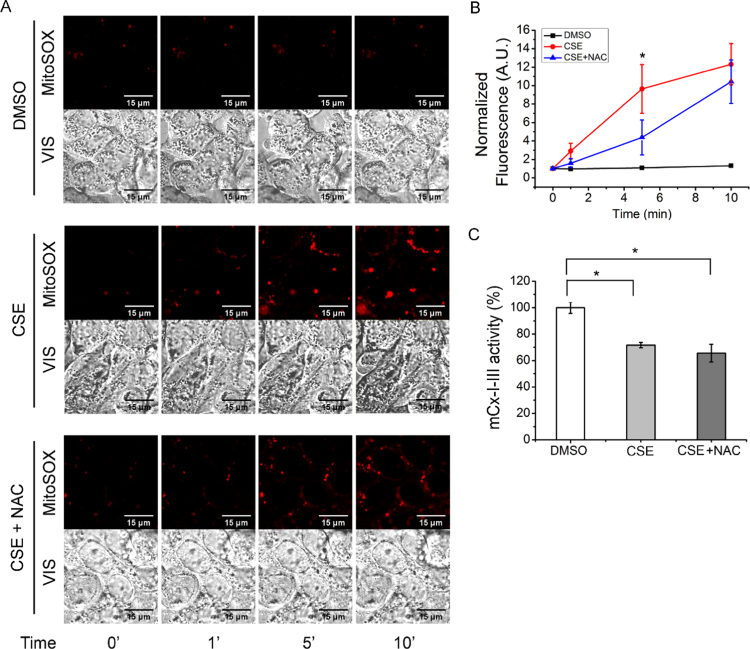
**Effects of CSE on mitochondrial ROS levels and Complex I-III activity.** A) Confocal microscopy corresponding to mitochondrial ROS (mtROS) levels measured by using MitoSOX at different times (0, 1, 5 and 10 min) in the presence of 100 µg/ml of CSE. Images were taken by using the time series configuration of the LSM 510 confocal microscope, a Plan-Neofluar 100×/1.3 Oil objective, a laser filter of 488 nm, and a LP filter of 560 nm. VIS indicates the visible image of cells. B) Normalized MitoSOX fluoresce values of corrected total cell fluorescence (CTCF) corresponding to the images shown in A. Fluorescence was normalized relative to control cells. C: Mitochondrial NADH-cytochrome c reductase (mCx-I-III) measured in Calu-3 cells incubated with 100 μg/ml CSE (CSE), DMSO or 100 μg/ml CSE + 5 mM NAC (CSE+NAC) for 24 h. Data were expressed as mean ± SEM of three independent experiments (n = 3). * indicates p < 0.05 compared to CSE-treated cells. Statistical analyses were performed by ANOVA and Tukey's test.

To study the effects of CSE over mitochondrial ROS levels, we used the mtROS probe MitoSOX and performed a live time-series analysis in the presence of vehicle, CSE (100 μg/ml) or CSE (100 μg/ml) CSE plus NAC (5 mM). As shown in Fig. 5A (photograph) and 5B (quantification of images fluorescence), it was observed a fast increase (within 5 min) in the mtROS production in cells exposed to CSE. The MitoSOX fluorescence intensity in cells treated with CSE plus NAC showed a partial attenuation by NAC after 5 min, which was not longer significant after 10 min (Fig. 5B). This reduction could be due in part to the presence of some MitoSOX in cytoplasm (since a high concentration of 5 µM MitoSOX was used) [43]. However, most of the signal is not reduced by NAC and this is more evident after 10 min. The lack of NAC effectiveness to reduce the CSE-induced mtROS suggests that NAC cannot reach mitochondria at levels enough to reduce mtROS.

High mtROS levels impair the correct functioning of the mitochondrial proteins and cause mtDNA damage by oxidation [15], [44], [45]. Thus, the impairment of the oxidative phosphorylation system (OXPHOS) by oxidation of some major components, such as the mitochondrial Complex I and III, might cause a vicious circle of ROS production that could extend the oxidative damage [15], [46]. To test if CSE exposure caused an impairment in the OXPHOS, the NADH-cytochrome c reductase (mCx-I-III) activity was measured. Mitochondria were isolated from Calu-3 cells incubated in the presence of vehicle, 100 μg/ml CSE or 100 μg/ml CSE plus 5 mM NAC for 24 h. As shown in Fig. 5C, the mCx-I-III activity decreased (p < 0.05, n = 3) in cells exposed to CSE as compared to control cells. Interestingly, NAC treatment was not able to block the effect of CSE on the mCx-I-III activity (Fig. 5C).

## 4 Discussion

**Fig. 6:**
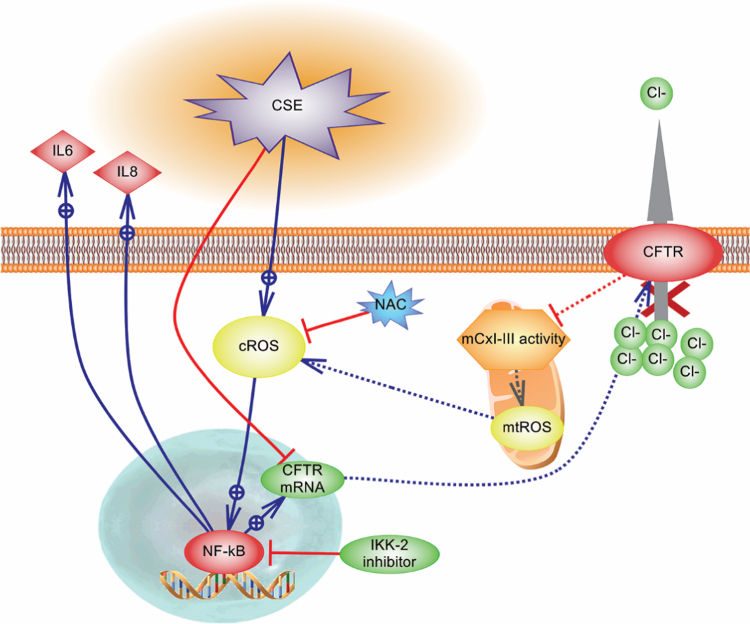
CSE effects on Calu-3 cells. The figure illustrates the effects of CSE in CFTR expression and activity, the mCx-I-III inhibition, the increased cROS and mtROS production, the pro-inflammatory response as IL-6 and IL-8 secretion, and the possible mechanisms involved. The cytoplasmic effects of CSE were reverted by incubations with NAC or NF-κB inhibitor (IKK-2 inhibitor). The NAC treatment inhibited cROS production whereas was not enough to effectively block mtROS production (green solid line -|) or to revert the mCx-I-III activity. Stimulations: blue line and arrow; inhibitions; red line and -|. The illustration shows the results obtained here (solid lines) and the postulated mechanisms (dotted lines). (For interpretation of the references to color in this figure legend, the reader is referred to the web version of this article.)

The aim of this work was study the effects of NAC treatment over the proinflammatory response produced by CSE in the epithelial airway cells Calu-3, used as a simplified *in vitro* model for COPD [25]. The results obtained are summarized in Fig. 6, together with the possible mechanisms involved. Using a CSE concentration and incubation time of 100 µg/ml during 24 h, no significant effects over cells survival were observed, and this concentration and time were used in subsequent experiments. *CFTR* mRNA expression and activity were significantly reduced after CSE exposure, in agreement with previous results from other laboratories in diverse model systems, showing deleterious effects of CSE on CFTR activity [1], [6], [7], [8], [9], [10], [11], [12], [47]. Thus, these results suggest that Calu-3 cells are a good model system for COPD studies.

Using this model system, we observed increased IL-6 and IL-8 levels after CSE exposure. Interestingly, this response was totally reverted by treatment with NAC, suggesting that a ROS signaling, induced by CSE, is involved in the production of these proinflammatory cytokines. This is in agreement with prior publications in other model systems [19], [48], [49], [50], [51]. It is important to highlight that the increased cytokine levels were induced directly by CSE, in absence of bacterial infection (aseptic or sterile inflammation) or LPS stimulation, as it has been proposed by other authors [19], [48]. Zhou et al. [51] identified different CSE compounds that could induce the production of IL-8 in human epithelium bronchial 16HBE cells, suggesting that the chemical composition of the CSE could start the inflammatory phenotype in COPD, previously to any bacterial infection. Also, Ko et al. observed that CSE exposure induces IL-8 release from macrophages mediated by ROS activation of NF-κB through the AMP-activated protein kinase (AMPK). NAC treatment also attenuated the IL-8 induction [49].

Measuring the DCF fluorescence, a significant increase in ROS production was observed in Calu-3 cells treated with CSE (100 µg/ml) for 1 h. Interestingly, NAC (5 mM) was able to completely block this effect, as occurred with the IL-6 and IL-8 secretion. On the other hand, the mitochondrial ROS (mtROS) production, measured by using MitoSOX, also increased very rapidly after the CSE treatment (in less than 5 min). The NAC treatment produced a partial attenuation in the MitoSOX signal, significant at 5 min, which could be in part due to some MitoSOX accumulated in the cytoplasm [43]. However, the NAC treatment was insufficient to completely block the induced mtROS at 5 min and after 10 min the differences were no longer significant.

In addition to the increased mtROS production, after 24 h of exposure to 100 µg/ml CSE, it was observed a decreased mCx-I-III activity that was not reverted by NAC exposure. These data suggest that this antioxidant treatment was not effective to block and protect key components of the mitochondrial OXPHOS (or that NAC by itself affects the mCx-I-III activity). Several reports suggest that the inhibition of mCx-I cause increased ROS levels in some diseases [52], [53]. In fact, it has been previously observed a reduced mCx-I activity and increased ROS production in cells with impaired CFTR activity [13], [14], [15], [27], [54]. The fast mtROS induction observed with CSE (< 5 min) suggests that the reduction in the CFTR activity and expression, as well as the reduction in the mCx-I-III activity, occur after the increase in ROS levels. However, the specific role of CFTR regarding the inflammatory response induced by CSE is not clear yet, since NAC could not revert the low CFTR levels induced by CSE, although it normalized the IL-6 and IL-8 levels. Thus, the reduced CFTR levels could be just a consequence of the CSE treatment, and not the cause for the increased IL-6 and IL-8 levels. Since the CFTR failure increases IL-1β levels and decreases mCx-I-III activity [13], [55], CFTR might have some role in the CSE-induced proinflammatory state and mitochondrial failure, that is not corrected by NAC treatment. In fact, NAC treatment also failed to normalize the CFTR levels. A CFTR correction, normalizing the mCx-I-III and ROS activities, might therefore be of clinical relevance for COPD treatment [56], [57], perhaps accompanied by NAC treatment. However, further research is needed to better understand the complex mechanisms involved in the CFTR down-regulation and signaling [27], [55], [58], [59], [60], [61], [62], [63], [64], [65], and particularly under CSE exposure.

We conclude that Calu-3 cells constitute an appropriate *in vitro* model to study the effects of CSE on oxidative and proinflammatory responses. A very rapid oxidative stress is developed under CSE exposure, which initiates a sterile proinflammatory response, affecting also the mitochondrial function, the CFTR levels, and causing changes in ROS production (illustrated in Fig. 6). NAC treatment could inhibit the CSE cytoplasmic effects on ROS and cytokines, although the mitochondrial effects on ROS were only partially reverted and NAC had no effects on the reduced mCx-I-III activity. Other inhibitors able to circumvent the mitochondrial failure and the low CFTR levels could be useful to avoid exacerbations in COPD.
