# Supplementary material for: N-acetyl cysteine reverts the proinflammatory state induced by cigarette smoke extract in lung Calu-3 cells
Source: Redox Biol. 2018 Mar 14;16:294–302. doi: 10.1016/j.redox.2018.03.006 (PMC5953002; doi:10.1016/j.redox.2018.03.006)
Supplement: Supplementary file 1 — Supplementary material [file mmc1.docx]

**Supplementary Information**


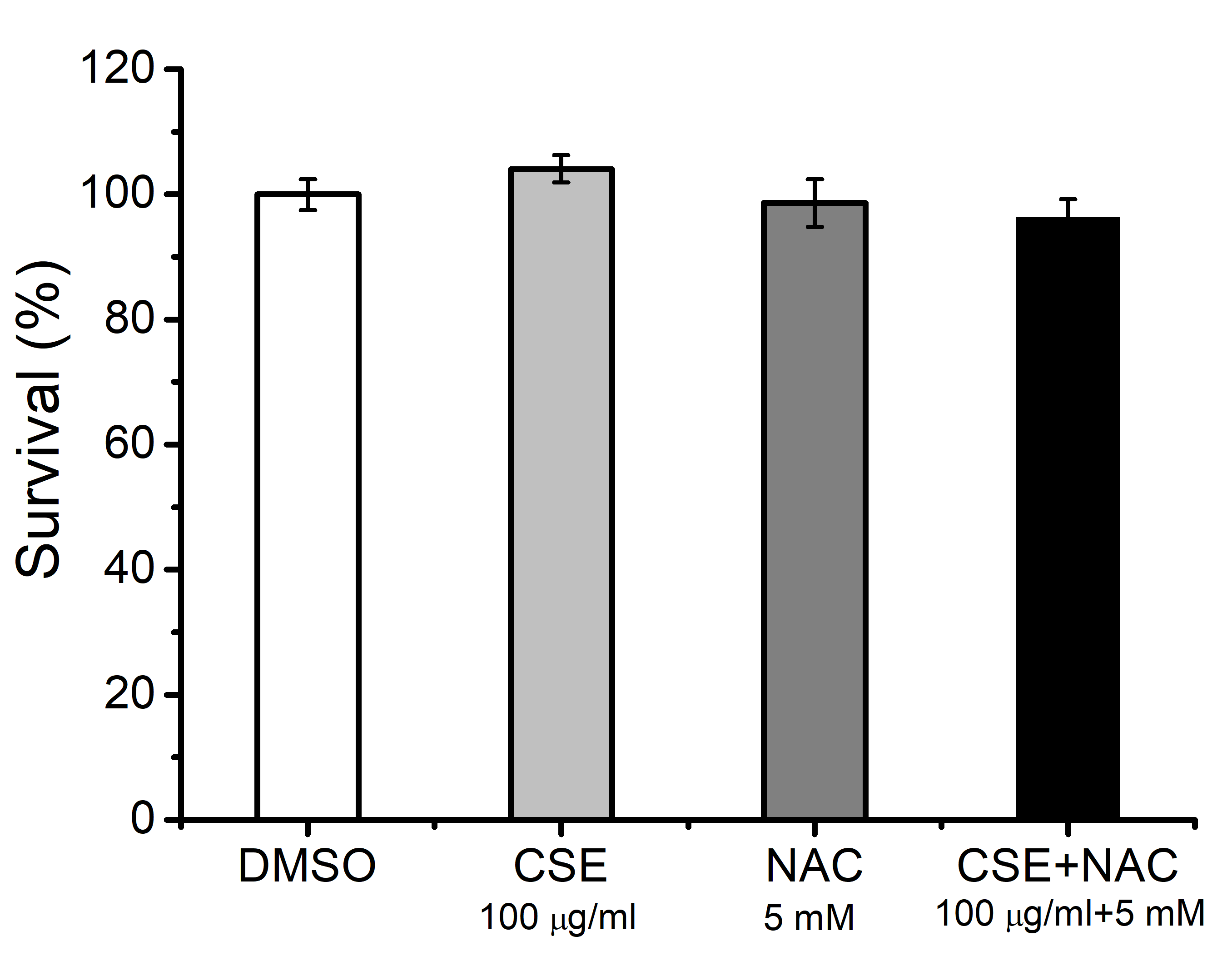


**Supplementary Figure 1: Cell viability in Calu-3 cells exposed to DMSO, CSE, NAC and CSE+NAC.** Cell viability for DMSO (0.25%, vehicle in all treatments), CSE exposure (100 µg/ml), NAC (5 mM) and CSE+NAC (100 µg/ml+5 mM respectively) exposure for 24 h). Viability was expressed as survival percentage (%) referred to control cells without CSE treatment (DMSO) as 100 %.
